# Emergency sclerotherapy for giant lymphatic malformation of the head and neck with airway obstruction in neonates

**DOI:** 10.3389/fped.2026.1805996

**Published:** 2026-04-01

**Authors:** Xin Zhang, Liang Wang, Jing Li, Zhuang Liu, Dan Song

**Affiliations:** 1 Department of Vascular Anomalies and Interventional Radiology, Children’s Hospital Affiliated to Shandong University, Jinan City, Shandong, China; 2 Department of Vascular Anomalies and Interventional Radiology, Jinan Children’s Hospital, Jinan City, Shandong, China

**Keywords:** airway obstruction, head and neck, lymphatic malformation, neonate, sclerotherapy

## Abstract

**Objective:**

To evaluate the efficacy and safety of emergency sclerotherapy for treating neonates with giant head and neck lymphatic malformations (LMs) complicated by airway obstruction.

**Methods:**

This retrospective analysis included the clinical data of neonates with giant head and neck LMs complicated by airway obstruction treated at the Children's Hospital Affiliated to Shandong University from January 2020 to December 2024. All patients underwent emergency sclerotherapy under general anesthesia and real-time ultrasound guidance. After percutaneous puncture and pigtail drainage catheter placement, lesions were treated with alternating lavage with pingyangmycin and 1% lauromacrogol. Lesion volume was measured using three-dimensional (3D) volumetric MRI, and therapeutic efficacy was evaluated–3–6 months after the final treatment according to the volume reduction rate.

**Results:**

A total of 13 neonates (8 males, 5 females) were enrolled in this study, with a mean birth weight of 3.47 ± 0.65 kg and a mean maximum lesion diameter of 9.42 ± 2.03 cm (range: 6.2–14.5 cm). There were seven cases of macrocystic LMs and six cases of mixed LMs, among which five lesions extended into the upper mediastinum and three had intralesional hemorrhage. A total of 57 sclerotherapy sessions were performed, with an average of 4.38 sessions per patient. The 3 neonates who underwent endotracheal intubation were successfully extubated 2–3 days after treatment. All patients achieved resolution of respiratory symptoms, with normal feeding and growth and no recurrence during follow-up. All 7 macrocystic LMs achieved excellent outcomes after a single sclerotherapy course. Among the six mixed LMs, four achieved excellent outcomes with one course, one required an additional sclerotherapy session, and one achieved excellent response after seven months of oral sirolimus. Transient low-grade fever (4 cases) and local swelling (7 cases) were observed as adverse reactions which resolved with symptomatic treatment, and no severe complications occurred.

**Conclusion:**

Emergency sclerotherapy combined with indwelling drainage catheters is an effective and safe treatment for neonates with giant head and neck LMs complicated by airway obstruction. The alternating use of sclerosing agents, together with adjunctive sirolimus, enhances efficacy with minimal adverse reactions, offering greater therapeutic benefit than to single-agent therapy and significant clinical value.

## 1 Introduction

Lymphatic malformations (LMs) are congenital low-flow vascular anomalies with an incidence of approximately 1.2–2.8 cases per 1,000 births, with nearly 90% presenting in children under 2 years of age (1). They consist of variably dilated lymphatic vessels or cysts lined by endothelium with a lymphatic phenotype and can be classified into macrocystic, microcystic, or mixed subtypes (2). LMs commonly occur in lymphatic-rich regions, particularly the head and neck. Infection, trauma, or intracystic hemorrhage can cause the acute enlargement of lesions, leading to compressive symptoms (3). Giant head and neck LMs pose a risk of life-threatening complications, including respiratory distress and dysphagia (4), due to increased pressure on the surrounding organs. In such cases, emergency interventions are required to preserve the patient's life.

Current treatment options for LMs are diverse, including surgical resection, sclerotherapy, ablation therapy, and systemic medical management. Surgical resection was once considered the preferred treatment, particularly for focal, large cystic, or life-threatening obstructive lesions. However, is associated with substantial morbidity, and a high complication rate (especially bleeding, iatrogenic injury, and local deformity), and uncertain efficacy (5). Recently, sclerotherapy has become the primary treatment option for LMs. Available sclerosants include anhydrous ethanol, bleomycin, pingyangmycin, polidocanol, lauromacrogol, OK-432, and doxycycline. Although sclerotherapy achieves good therapeutic outcomes and offers advantages such as minimal invasiveness and reduced pain, the side effects of sclerosing agents in newborns cannot be overlooked.

To enhance the efficacy of sclerotherapy while minimizing its adverse effects, this study summarizes our clinical experience with the emergency treatment of neonates with giant head and neck LMs complicated by airway obstruction, to provide a safe and effective therapeutic reference for this critical population.

## 2 Methods

### 2.1 Patients and clinical data

A retrospective study was conducted on neonates with giant head and neck LMs complicated by airway obstruction who were treated at the Department of Vascular Anomalies and Interventional Radiology, Children's Hospital Affiliated to Shandong University, from January 2020 to December 2024. The inclusion criteria were: (1) Neonatal stage (≤28 days after birth) at the time of diagnosis; (2) A confirmed diagnosis of head and neck LMs based on imaging findings (ultrasound and MRI); (3) the presence of airway obstruction with clinical symptoms such as respiratory distress; (4) receipt of emergency sclerotherapy as the primary treatment. The exclusion criteria were as follows: (1) concomitant other severe congenital malformations or organic diseases; (2) coagulation dysfunction, severe infection, or drug allergy contraindicating sclerotherapy; and (3) incomplete clinical and follow-up data.

All enrolled patients underwent routine ultrasound and MRI examinations as well as preoperative blood tests, coagulation profiles, liver and kidney function tests, and infection marker assessment to exclude treatment contraindications.

This study was approved by the hospital ethics committee, and written informed consent was obtained from the parents for the use of clinical data and publication of relevant medical images.

### 2.2 Preparation of sclerosing agents

To prepare pingyangmycin, 8 mg of pingyangmycin (Yanji Company, Jilin Jiaodong Pharmaceutical Group) was dissolved in 4 mL of ioxaglate (Shanghai GE Healthcare Pharmaceuticals Co., Ltd.), and then 1–2 mg of dexamethasone was added and mixed thouroughly for use.

Lauromacrogol was used in its undiluted form at a concentration of 1% (100 mg/10 mL; Shanxi Tianyu Pharmaceutical Co. Ltd.).

### 2.3 Treatment methods

All patients underwent urgent sclerotherapy under general anesthesia. Under real-time color Doppler ultrasound guidance, percutaneous lesion puncture was performed using an 18G pediatric-specific cannula (Tenlmo, Tokyo, Japan). Successful puncture was indicated by the aspiration of pale-yellow lymphatic fluid from the cannula tip. If bloody fluid appeared at the needle tip, immediate assessment of coagulability was required. If the fluid clotted, indicating accidental vascular puncture, the procedure was immediately terminated. After needle removal, compression hemostasis was performed and a new puncture site was selected. After successful cystic cavity puncture, the needle core was withdrawn. A 0.035-inch stiffened guidewire was advanced through the outer sheath and secured within the cystic cavity. Subsequently, the outer sheath was withdrawn. The puncture pathway was dilated along the guidewire using a dilator. After dilation, a Cook's pigtail drainage catheter was inserted over the guidewire. The contrast medium was injected through the catheter, and digital subtraction angiography (DSA) fluoroscopy was performed to confirm that the catheter tip was fully positioned within the cyst cavity. Thereafter, the drainage catheter was fixed. In lesions with multiple large cystic compartments, multiple drainage catheters could be placed simultaneously to ensure therapeutic efficacy.

During treatment, the residual lymphatic fluid within the cystic cavity was aspirated daily using a syringe. Alternating intralesional injections of pingyangmycin and lauromacrogol were administered through the drainage catheter. After each injection, the solution was allowed to stand for 20 min. If necessary, the patient's position was adjusted to ensure adequate contact between the solution and the cyst wall. Subsequently, most of the residual solution was aspirated from the cystic cavities. Once the daily drainage volume decreased to ≤5 mL, the drainage catheter was removed. Prior to removal, a single dose of pingyangmycin solution (calculated at 8–10 mg/m^2^ body surface area per treatment course) was administered into the cystic cavity and retained without aspiration. Enhanced infection control measures were implemented during catheter placement, with regular disinfection of the puncture site. For patients with residual macrocystic LMs on imaging one month after catheter removal, repeat sclerotherapy was indicated. Reassessment was conducted one month later to determine the need for further treatment.

For residual microcystic lesions in mixed LMs following sclerotherapy, oral sirolimus therapy was initiated at a dose of 0.8 mg/m^2^ per administration every 12 h, with close monitoring of serum drug concentrations and adverse drug reactions.

### 2.4 Evaluation of therapeutic effect

#### 2.4.1 Imaging evaluation

The efficacy of sclerotherapy was assessed by physical examination and three-dimensional (3D) volumetric MRI at 3–6 months after the final treatment session. T2-weighted MRI scans were performed before and after treatment, and the lesion volume was measured using standardized three-dimensional (3D) volumetric analysis software (Philips IntelliSpace Portal, Netherlands). The region of interest (ROI) was manually delineated slice-by-slice along the lesion boundary on all axial, coronal, and sagittal images to ensure complete coverage of the entire lesion, and the total lesion volume was calculated by summing the areas of the ROIs across all slices and multiplying by the slice thickness. The volume reduction rate was calculated using the formula: Volume reduction (%) = (Vpre - Vpost)/Vpre  ×  100%, where Vpre is the pre-treatment lesion volume and Vpost is the post-treatment lesion volume.

#### 2.4.2 Efficacy grading criteria

Based on the imaging-measured volume reduction rate at 3–6 months after the final sclerotherapy session, the treatment outcomes were categorized into four grades (6): (1) Excellent response: ≥80% volume reduction; (2) Moderate response: 50%–80% volume reduction; (3) Mild response: 10%–50% volume reduction; (4) No response: <10% volume reduction.

#### 2.4.3 Clinical efficacy evaluation

In addition to imaging indicators, clinical efficacy was evaluated during the follow-up on the basis of the following parameters: (1) relief of airway obstruction symptoms (disappearance of tachypnea, dyspnea, cyanosis, etc.); (2) recovery of oral feeding function (resumption of normal feeding without dysphagia or aspiration); and (3) neonatal weight gain and growth and development status [assessed by the Denver Developmental Screening Test (DDST)].

#### 2.4.4 Safety evaluation

The safety of the treatment was evaluated by recording the occurrence of adverse reactions during and after treatment, including transient low-grade fever, local swelling, secondary infection, drug allergy, and pulmonary fibrosis and by recording the type, number of cases, and resolution of adverse reactions.

## 3 Results

### 3.1 Baseline clinical characteristics of patients

A total of 13 neonates were enrolled in this study, including 8 males and 5 females, with a birth weight ranging from 2.5 to 4.8 kg and a mean of 3.47 ± 0.65 kg. The patients' age at presentation ranged from 1 to 21 days after birth. Imaging examinations confirmed that the maximum diameter of the lesions ranged from 6.2 to 14.5 cm, with a mean of 9.42 ± 2.03 cm; 8 cases of lesions were limited to the head and neck region, and 5 cases involved both the head/neck and upper mediastinum; all lesions caused tracheal compression and luminal narrowing, including 7 cases of macrocystic LMs and 6 cases of mixed LMs; 3 cases were associated with intralesional hemorrhage at initial diagnosis, and 3 cases had severe respiratory failure requiring endotracheal intubation for assisted ventilation at admission (Table 1).

**Table 1:** Clinical characteristics of the patients.

Case	Gender	Age at Consultation (days)	Body Weight (kg)	Site	Type	Diameter (cm)	Compression	Number of irrigation-sclerotherapy sessions	Complications
1	F	4	2.87	Head and Neck	Macrocystic	8.9	Trachea	4	Fever, Swelling
2	M	1	3.30	Neck and Mediastinum	Macrocystic	8.4	Trachea	5	Swelling
3	M	5	3.43	Head and Neck	Mixed	11.2	Trachea	5	
4	M	2	3.45	Head, Neck, and Mediastinum	Macrocystic	14.5	Trachea and Esophagus	4	Swelling
5	F	15	3.82	Head and Neck	Mixed	7.4	Trachea	3	
6	M	13	4.67	Neck	Mixed	10.2	Trachea	4	Swelling
7	F	9	2.98	Head and Neck	Mixed	9.1	Trachea	5	Fever
8	F	1	2.50	Head, Neck, and Mediastinum	Macrocystic	6.2	Trachea	5	Fevers, Swelling
9	M	2	3.15	Head and Neck	Mixed	9.5	Trachea	4	
10	M	21	4.80	Head and Neck	Macrocystic	8.8	Trachea	4	Swelling
11	M	4	3.56	Head, Neck, and Mediastinum	Mixed	7.9	Trachea	5	
12	F	3	3.28	Head, Neck, and Mediastinum	Macrocystic	10.2	Trachea	6	Fever, Swelling
13	M	3	3.30	Head and Neck	Macrocystic	10.1	Trachea	3	

### 3.2 Treatment and follow-up status

All 13 neonates successfully completed emergency sclerotherapy, with 57 sclerotherapy sessions performed after drainage catheter placement, for an average of 4.38 sessions per patient. The 3 neonates who underwent endotracheal intubation were successfully extubated 2–3 days after drainage catheter placement and sclerotherapy. All patients were followed up at 1, 3, and 6 months after the final treatment, with a median follow-up duration of 4.5 months, and no loss to follow-up occurred.

### 3.3 Therapeutic efficacy

#### 3.3.1 Relief of clinical symptoms

All patients achieved complete relief of airway obstruction symptoms (tachypnea, dyspnea, and cyanosis) at the 1-month follow-up, and no recurrence of airway obstruction was observed during the entire follow-up period. All neonates resumed full oral feeding within 1 week after treatment, with no cases of feeding difficulties or aspiration; the mean weight gain at the 6-month follow-up was 1.82 ± 0.45 kg, which was within the normal range for their corrected age. DDST at the 6-month follow-up showed normal growth and development in all patients, with no developmental delays observed.

#### 3.3.2 Imaging efficacy outcomes

**Figure 1:**
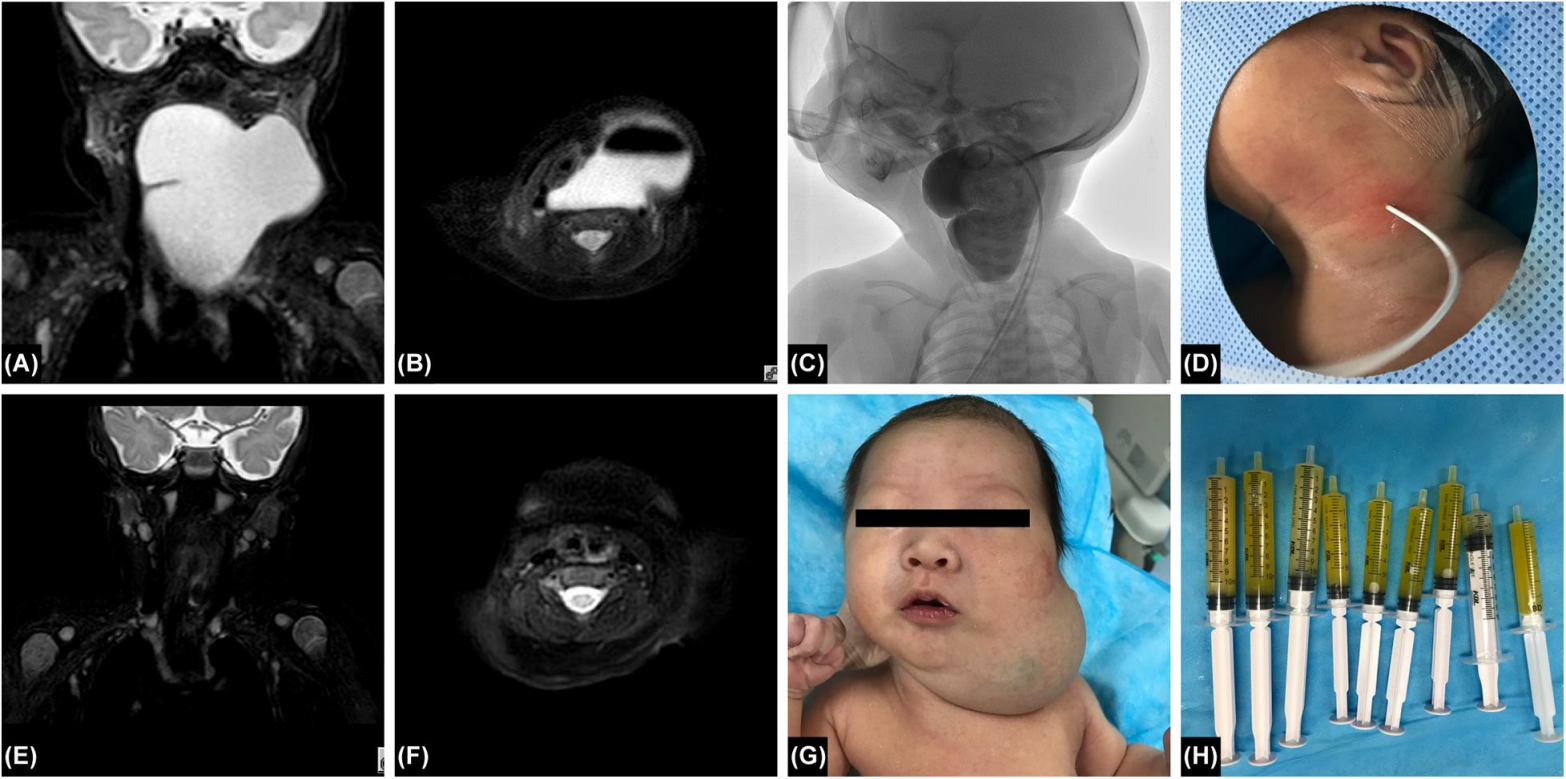
**(A)** Pre-treatment coronal T2-weighted MRI image showing a large, well-defined hyperintense lesion in the head and neck region, suggestive of lymphatic malformation with cystic fluid accumulation. **(B)**: Pre-treatment T2-weighted MRI image showing a large lesion compressing surrounding tissues. **(C)**: Intracavitary view after percutaneous puncture during treatment, with pigtail drainage catheter in place. DSA single-frame fluoroscopic image demonstrating contrast agent diffusion within the lesion cavity, clearly delineating its morphology and extent. **(D)**: Intraoperative view showing the puncture needle placement site for percutaneous insertion into the lesion cavity. **(E)**: Follow-up coronal T2-weighted MRI after pingyangmycin and 1% lauromacrogol alternating sclerotherapy shows significant reduction in lesion volume, near-complete disappearance of the hyperintense cystic cavity, and normalization of residual tissue signal. **(F)**: Post-sclerotherapy T2-weighted MRI demonstrates near-complete resolution of the lesion with clear visualization of surrounding tissues. **(G)**: Pre-treatment clinical photograph showing diffuse swelling of the head and neck, with facial deformity due to lesion compression. **(H)**: Pale yellow lymphatic fluid aspirated.

**Figure 2:**
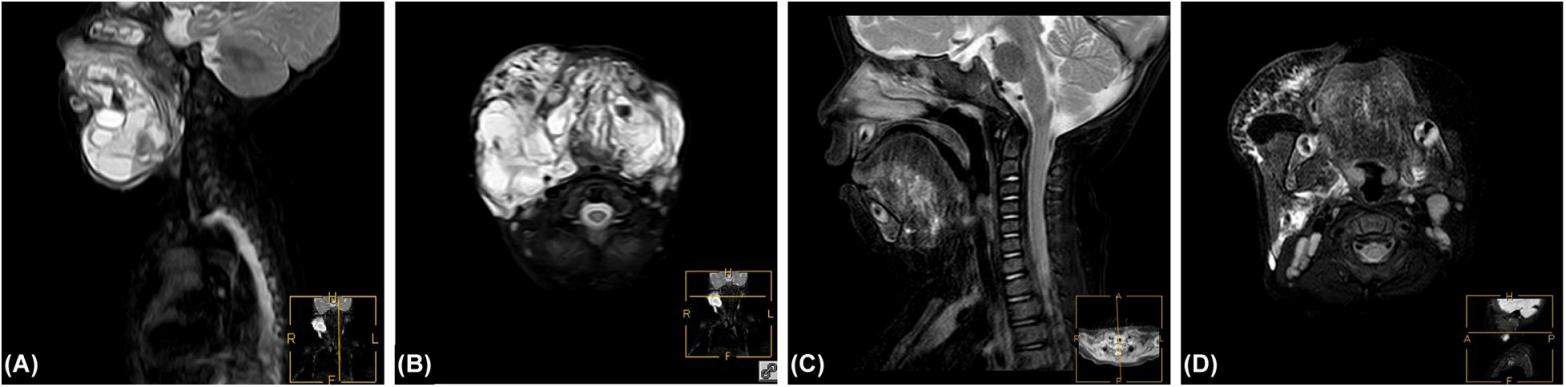
**(A)**: Pre-treatment MRI sagittal fat-suppressed image shows a mixed-signal mass in the head and neck region, suggestive of a hemorrhagic lymphatic malformation. **(B)**: Pre-treatment MRI axial fat-suppressed T2-weighted image reveals an irregular, heterogeneous hyperintense lesion in the head and neck with patchy hemorrhagic signal patterns. The lesion has indistinct margins and causes displacement of surrounding soft tissues. **(C)**: Post-treatment sagittal fat-suppressed T2-weighted MRI after initial pingyangmycin and 1% lauromacrogol alternating sclerotherapy followed by 7 months of oral sirolimus adjuvant therapy shows significant volume reduction of the original lesion with more uniform signal intensity and markedly alleviated compression of surrounding tissues. **(D)**: Post-treatment axial fat-suppressed T2-weighted MRI demonstrates a markedly reduced area of the original mixed-signal lesion with near-complete resolution of abnormal hyperintensity, revealing clear anatomical structures including surrounding muscles and vessels.

According to the efficacy grading criteria, all seven patients with macrocystic LMs achieved excellent responses after a single sclerotherapy course and required no further treatment after catheter removal (Figure 1). Among the six patients with mixed LMs, four achieved excellent responses after one sclerotherapy course with no additional treatment needed, one achieved an excellent response after one additional sclerotherapy session, and one patient with diffuse microcystic residual lesions and maxillofacial deformity after sclerotherapy achieved an excellent response after seven months of oral sirolimus adjunctive therapy (Figure 2). The overall response rate was 100%.

### 3.4 Safety and adverse reactions

During the entire treatment and follow-up period, no severe adverse reactions such as secondary infection, drug allergy, or pulmonary fibrosis were observed in any patient. The observed treatment-related adverse reactions were mild and transient, including four cases of transient low-grade fever and seven cases of local swelling at the puncture/lesion site, all of which resolved completely with symptomatic management such as antipyretics and local care, without affecting the continuation of treatment or follow-up.

## 4 Discussion

LMs are vascular anomalies resulting from developmental abnormalities of the embryonic lymphatic system development. Histologically, they comprise multiple dilated small lumens or cystic structures lined with a single layer of endothelial cells and supported by connective tissue stroma, interspersed with lymphoid follicular cells, and occasional germinal centers (7). Approximately 75% of cases occur in the head and neck region, where lymphoid tissues are densely concentrated (8). The complex anatomical relationship between these lesions and adjacent airway structures may pose life-threatening risks. Some cases are detected during fetal development, and reports indicate an incidence of 0.6% of fetal LMs between 11 and 14 weeks of gestation, with a spontaneous abortion rate of approximately 1 in 750 in such fetuses (9). In one of our cases, fetal ultrasound at 34 weeks of gestation revealed head and neck LM without significant intrauterine distress. Serial follow-up ultrasonography during pregnancy revealed no marked lesion enlargement. After birth, physical examination demonstrated marked enlargement of the head and neck mass relative to prenatal imaging, accompanied by tachypnea. Sclerotherapy was performed urgently due to suspected intralesional hemorrhage.

Treatment of LMs typically aims to restore or preserve organ function or address cosmetic concerns. Both surgical excision and sclerotherapy can achieve local lesion control and relieve symptoms. Treatment decisions depend on the location and size of the malformation, the extent of adjacent tissue involvement, and the treating physician's clinical experience (10). Surgical intervention can rapidly remove lesions and relieve airway compression. However, complete surgical resection is often challenging because of the infiltrative nature of LMs. Surgical complications include damage to surrounding vital structures (including vascular or neural injury), incomplete resection secondary to lesion infiltration near critical anatomical structures, prolonged wound lymphatic drainage, and surgical site infection. Previous reports have shown a significantly increased risk of surgical complications for LMs in patients under 12 months of age, and in those with head and neck lesions (11). With the advancement of sclerotherapy techniques and the introduction of novel sclerosing agents, surgery is no longer the preferred first-line treatment in many clinical scenarios. Sclerotherapy is an effective treatment modality for head and neck LMs with a reported clinical success rate of 84% (12). This procedure involves percutaneous puncture of the lesion to inject sclerosing agents into the cystic cavity, thereby destroying the endothelial lining of the lymphatic malformation, and inducing localized fibrosis to ultimately achieve cyst lumen occlusion. Sclerotherapy is typically performed under real-time imaging guidance (e.g., ultrasonography or DSA).

Common sclerosing agents include bleomycin, pingyangmycin (13), polidocanol, lauromacrogol, OK-432, and anhydrous ethanol. Absolute ethanol is a highly effective sclerosant used for high-flow vascular malformations, and has also shown favorable therapeutic efficacy in treating low-flow vascular malformations. Zhang et al. confirmed that the efficacy of absolute ethanol in the treatment of low-flow vascular malformations was significantly superior to that of bleomycin (14). However, absolute ethanol is typically used in experienced centers, and there are relatively few reports on its application in a special population of neonates with lymphatic malformations. Therefore, absolute ethanol was not selected for use in this study. OK-432 is a freeze-dried culture composed of Group A pyogenic streptococci of human origin. Its clinical efficacy is achieved by damaging the endothelial cells of LMs, through activation of the immune system (macrophages, NK cells, and LAK cytotoxic T lymphocytes). Consequently, the initial injection induces a robust inflammatory response accompanied by swelling, erythema, pain, and fever that persist for several days. These pronounced systemic inflammatory reactions ultimately limit the use of OK-432 in neonates and young infants with immature immune systems (15). Pingyangmycin is an antitumor antibiotic extracted from the culture medium of Streptomyces pingyangensis isolated from the soil in Pingyang, Zhejiang Province, China. It belongs to the bleomycin class of antitumor drugs and shares a chemical structure highly similar to that of Bleomycin A5 (internationally recognized standard denomination). It is widely used for sclerotherapy of vascular malformations (16–18). However, it is still associated with complications, including local swelling, hyperpigmentation, fever, allergic reactions, and pulmonary toxicity (19). Lauromacrogol, similar to polidocanol, is a sclerosing agent widely used in cyst treatment. Recent reports have indicated its use in LMs with acceptable efficacy and fewer complications (20). As a special population, neonates have underdeveloped hepatic and renal functions, necessitating strict dosage restrictions for sclerosing agents. For giant head and neck lesions, the dosage of sclerosing agents is relatively insufficient and may be diluted by accumulated lymph fluid, potentially leading to suboptimal sclerotherapy outcomes and requiring multiple treatment sessions. We administered alternating intralesional injections of two sclerosing agents (pingyangmycin and lauromacrogol) to minimize excessive single-agent dosing and prevent prolonged drug retention within the cyst cavity.

A pigtail drainage catheter was placed within the cystic cavity to achieve rapid decompression of airway obstruction. The distal end of the drainage catheter contained multiple side holes to facilitate complete drainage of lymphatic fluid from multicompartment cystic lesions. Drainage catheter placement was guided by ultrasound, with the tip positioned in the largest cyst or in the compartment causing the most prominent compression on the airway. After placement, a small amount of contrast medium was injected and the catheter position was confirmed using DSA fluoroscopy. When adjustment was required, single-frame fluoroscopy was used to minimize ionizing radiation exposure in neonates. Another key purpose of maintaining the drainage catheter *in situ* is to facilitate subsequent sclerotherapy sessions. In clinical practice, cystic lymphatic fluid was aspirated daily, followed by intralesional injection of sclerosing agents that are retained for 20 min. During this retention period, frequent positional adjustments were implemented to facilitate adequate contact of the sclerosants with the cyst wall endothelium, thereby enhancing treatment efficacy. Once the daily drainage volume decreased to <5 mL, a therapeutic dose of pingyangmycin was injected and retained without subsequent aspiration, followed by removal of the drainage catheter.

In our study, all patients with macrocystic LMs achieved an excellent response after a single session of sclerotherapy, and all patients with mixed-type LMs attained excellent outcomes following individualized additional treatment, resulting in an overall response rate of 100% among the 13 neonates. This therapeutic efficacy is markedly superior to the findings of previous meta-analyses on percutaneous sclerotherapy for LMs: a comprehensive meta-analysis of 25 studies involving 726 patients reported an overall complete cure rate of 50.5% (95% CI: 36.6%–64.3%) for all LM subtypes, with macrocystic lesions achieving a cure rate of 53.1%, while microcystic and mixed lesions had significantly lower rates of 35.1% and 31.1%, respectively (13). In contrast, our regimen of alternating dual sclerosants combined with indwelling pigtail drainage catheters achieved a 100% response rate, highlighting the clinical superiority of our strategy. Notably, two patients with mixed-type LMs required additional interventions, including re-sclerotherapy or oral sirolimus therapy, to optimize their outcomes. Although the single-course cure rate for mixed-type LMs was lower than that for macrocystic LMs, the short-term therapeutic goal of promptly relieving airway obstruction in neonates was successfully achieved in all mixed-type cases.

The outstanding efficacy of our regimen is mainly attributed to two core advantages: first, the alternating use of pingyangmycin and lauromacrogol exerts a synergistic sclerosing effect—pingyangmycin damages lymphatic endothelial cells by inducing DNA strand breakage, while lauromacrogol destroys endothelial cells through membrane-disruptive effects. Their complementary mechanisms may therefore produce more complete injury to the cyst wall endothelium and enhance the sclerotherapeutic effect. At the same time, alternating administration reduces exposure to any single agent, thereby limiting drug accumulation and potentially decreasing the risk of adverse effects, including pingyangmycin-related pulmonary toxicity. Second, the indwelling pigtail drainage catheter achieves rapid and thorough drainage of cystic fluid, which immediately relieves airway compression and is critical for successful emergency management of neonates with severe airway obstruction; in addition, the catheter provides a stable drug delivery channel for sequential sclerotherapy, avoiding multiple percutaneous punctures, reducing trauma and infection risks, and ensuring the effective concentration of sclerosing agents in the cyst cavity by draining the accumulated lymph fluid, thus improving the therapeutic effect.

Sirolimus, a mammalian target of rapamycin (mTOR) inhibitor, was initially used to prevent organ transplant rejection. Subsequent clinical studies have confirmed its significant efficacy in the management of congenital vascular malformations, establishing it as a key therapeutic agent for refractory LMs (21–23). Approximately 90% of patients with LMs demonstrate a favorable clinical response to sirolimus (3). One patient developed residual lesions dominated by microcystic components after sclerotherapy. Sequential oral sirolimus therapy was administered for 7 months, achieving an 80% reduction in lesion volume. No significant abnormalities were observed in hematological parameters, coagulation profiles, or hepatic and renal function indices, and no complications such as oral mucositis or infection occurred.

The limitations of the present study include a relatively small sample size and the absence of a control group, which limits the generalizability of our findings. Additionally, this was a single-center retrospective analysis without long-term follow-up data, making it impossible to evaluate the long-term efficacy and safety of the combined sclerotherapy regimen in neonates with head and neck LMs. Furthermore, standardized optimal criteria for sclerosing agent selection and dosage titration in this specific population remain undefined. Given the favorable therapeutic efficacy of absolute ethanol in the treatment of vascular malformations, future studies should explore the efficacy and safety of absolute ethanol in neonates with head and neck LMs to provide more therapeutic options for this population.

## 5 Conclusion

Sclerotherapy appears to be an effective therapeutic modality for neonates with massive head and neck LMs complicated by airway obstruction. The combination of alternating dual sclerosants and indwelling pigtail drainage catheters achieved favorable therapeutic outcomes with minimal adverse reactions, while adjuvant sirolimus may provide additional benefit in mixed LMs, supporting its value as a clinical option for this critical population.

## Data Availability

The original contributions presented in the study are included in the article/Supplementary Material, further inquiries can be directed to the corresponding author.
